# A Dynamically Correlated Network Model for the Collective Dynamics in Glass-Forming Molecular Liquids and Polymers

**DOI:** 10.3390/polym13193424

**Published:** 2021-10-06

**Authors:** Takashi Sasaki, Yuya Tsuzuki, Tatsuki Nakane

**Affiliations:** Department of Materials Science and Engineering, University of Fukui, Fukui 9108507, Japan; bash756@gmail.com (Y.T.); nakane19970319@gmail.com (T.N.)

**Keywords:** dynamics, supercooled liquids, dynamically correlated network, relaxation time, simulation

## Abstract

The non-Arrhenius behavior of segmental dynamics in glass-forming liquids is one of the most profound mysteries in soft matter physics. In this article, we propose a dynamically correlated network (DCN) model to understand the growing behavior of dynamically correlated regions during cooling, which leads to the viscous slowdown of supercooled liquids. The fundamental concept of the model is that the cooperative region of collective motions has a network structure that consists of string-like parts, and networks of various sizes interpenetrate each other. Each segment undergoes dynamical coupling with its neighboring segments via a finite binding energy. Monte Carlo simulations showed that the fractal dimension of the DCNs generated at different temperatures increased and their size distribution became broader with decreasing temperature. The segmental relaxation time was evaluated based on a power law with four different exponents for the activation energy of rearrangement with respect to the DCN size. The results of the present DCN model are consistent with the experimental results for various materials of molecular and polymeric liquids.

## 1. Introduction

The mechanism of glass formation in supercooled liquids is one of the most fundamental but unsolved issues in soft matter physics [1,2]. Glass-forming liquids generally exhibit a drastic increase in viscosity upon being cooled, which leads to an apparent freezing of molecular or segmental rearrangements observed as a laboratory glass transition phenomenon. The temperature dependence of the viscosity does not follow the Arrhenius law, which is an essential feature for liquids in a supercooled state. The origin of this non-Arrhenius behavior is still unclear, although extensive studies, including experiments, theories, and simulations have been conducted so far [3,4,5,6,7,8]. The glass transition phenomenon observed on the laboratory time scale is not considered a real thermodynamic transition; quite a few researchers assume that there exists an ideal transition in equilibrium: the apparent glass transition is a signature of this ideal phase transition [9,10,11]. The major theories in this aspect are based on this idea, which accords with the argument that, at a certain low temperature, some singularity is required to avoid the Kauzmann entropy crisis [12]. In contrast, other researchers consider the glass transition as a kinetic transition, i.e., a non-equilibrium phase transition without any singularities in thermodynamics (kinetically constrained model) [13,14,15]. Furthermore, theories of phase transitions based on dynamic facilitation were developed [16].

Notably, the viscous slowdown was successfully explained by cooperatively rearranging for α-relaxation, as proposed by Adam and Gibbs [9]. The length scale of this cooperativity (dynamic length scale) increases on cooling in supercooled liquids. The cooperativity leads to the viscous slowdown, which usually appears well above the ideal transition temperature (thermodynamic phase transition). In addition, each of the above cooperative regions generally has different molecular mobilities; thus, there exists spatial heterogeneity in dynamics [1,17,18,19]. Notably, the heterogeneous dynamics do not accompany any heterogeneities in the static structure at the same length scale. Thus, the origin of the spatial heterogeneity in dynamics is not directly related to the static structural features. The reason why supercooled liquids are dynamically heterogeneous is likely to be relevant to their non-Arrhenius behavior, although its origin is not yet elucidated.

A major concept for understanding the dynamics of supercooled liquids is that configurational entropy plays a significant role, as in the Adam–Gibbs approach. The random first-order transition (RFOT) theory, which is also in line with the entropy-based approach, was developed from the mean field theory of spin glasses, where the collective rearrangements are regarded as the transition between metastable configurational states [10,11,20,21,22,23]. The transition barrier is related to the surface and bulk free energies of the cooperative region, leading to a scaling relationship between the correlation length scale and the configurational entropy. Recent simulation studies have suggested that dynamic cooperative regions have string-like shapes rather than compact ones [24,25,26]. This may be inconsistent with the earlier picture of a mosaic structure for the cooperatively rearranging regions of the Adam–Gibbs theory. The string-like or non-compact shape may be a promising idea that can explain the inconsistency between the length scales of the dynamic and static heterogeneities. Furthermore, some simple phenomenological models were proposed, in which cooperative regions with non-compact shapes were assumed [27,28,29]. In addition, such phenomenological approaches were extended to explain the size-dependent dynamics in nanosized polymeric materials [30,31,32]. However, the origin of the anomalous dynamics and decoupling between quasi-thermodynamic and dynamic observables in nano-confined systems remains a controversial issue in polymer physics [33,34,35].

In this paper, we present a simple model that can reproduce the temperature dependence of segmental dynamics in supercooled liquids based on string-like collective dynamics. Segments undergo cooperative rearrangements via dynamical coupling with their neighboring segments, resulting in a dynamically correlated network (DCN) composed of string-like parts (strands). It is assumed that the DCN once formed persists until the cooperative rearrangement in it is completed. The relaxation time relevant to the rearrangement in the DCN depends on its size (the number of segments included in the DCN). It is also assumed that the DCN disappears immediately after the rearrangement is completed, i.e., the lifetime of a DCN corresponds to the relaxation time of the rearrangement. We executed Monte Carlo simulations to generate numerous DCNs based on the probabilities of dynamical coupling with neighboring segments. The size and size distribution of the generated DCNs were evaluated with respect to temperature, and the temperature dependence of the segmental relaxation time was evaluated. The results were compared with the experimental data for molecular liquids and polymers. The temperature dependence of the size distribution and geometry of the DCN was also investigated.

## 2. Modeling of DCN

In the DCN model, we first consider that a glass-forming liquid consists of a collection of identical segments that can rearrange cooperatively with other segments via dynamical coupling. A segment corresponds to a molecule in the case of molecular liquids or a motional unit in the case of polymeric liquids. The segments undergo rearrangement by forming a cluster with a network structure. The segments are placed at lattice points such that each segment has *z* neighboring segments (*z* is the coordination number). The neighboring *z* segments are assigned to be “correlated” or “uncorrelated” according to energy fluctuations: the “correlated” segments undergo cooperative rearrangements with the segment of interest, whereas the “uncorrelated” segments do not. Each segment is adjacent to a different number of “correlated” segments *n*, which obeys 1 ≤ *n* ≤ *z*. Here, we assume that dynamical coupling between the segments occurs via the coupling energy *ε*. The probability that a segment is adjacent to *n* correlated segments is proportional to the Boltzmann factor as follows:

*P_n_* = ∝ *_z_*C*_n_* exp[−(*z* − *n*)/T*][1 − exp(−1/T*]^*n*^ (1)

where *_z_*C*_n_* = *z*!/[*n*! (*z* − *n*)!], 0≤n≤z , and T* is the reduced temperature that is scaled by *ε* as

T* = *kT*/*ε* (2)

Here, *k* is the Boltzmann constant and *T* is the absolute temperature. The probabilities with respect to the temperature calculated from Equation (1) with *z* = 6 are shown in Figure S1 in Supplementary Materials.

**Figure 1:**
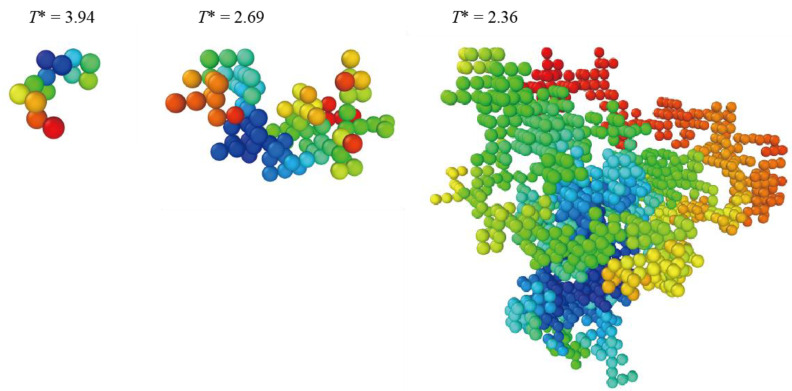
Examples of dynamically correlated networks (DCNs) generated via Monte Carlo simulation. The color of the segments varies gradually from blue to red in the order of being added to the network.

To generate DCNs, we performed Monte Carlo simulations, where the segments are correlated according to the probabilities of Equation (1). Here, we employed a simple cubic lattice with *z* = 6. First, a segment with a coupling number *n* was generated. Next, the neighboring *n* segments selected randomly were assigned to be “correlated” and the remaining *z* − *n* segments were assigned to be “uncorrelated”. Then, one of the “correlated” segments that has unassigned neighbors was selected randomly, and its neighbors were assigned in the same way as above. The procedure was repeated until there were no unassigned neighboring segments; thus, a completed DCN composed of *N* correlated segments was obtained. The networks thus obtained correspond to lattice animals on a type of Bethe lattice with *z* = 6 [36,37] under the condition that their growth is constrained as loops are allowed on a simple cubic lattice. We performed the simulations by using a sequential process as described below, which resulted in a distribution of *n* that differs from that prescribed by Equation (1). When the neighboring segments were assigned as either “correlated” or “uncorrelated”, the value of *n* was determined according to Equation (1) as a general rule. However, if there were values of *n* that were inhibited by the geometrical limitation of the lattice (there was a possibility that the neighbors were already assigned), the inhibited values of *n* were excluded from the possible choices. We generated at least 10^5^ DCNs at each temperature. Figure 1 shows examples of the generated DCNs.

The generated DCNs in the simulation did not include segments with *n* = 0. However, in a real system the possibility of an isolated segment (i.e., DCN with *N* = 1) should not be excluded as it has a finite probability according to Equation (1), although the probability is very low in the deeply supercooled range. Therefore, we took into account the contribution of the DCNs with *N* = 1 based on Equation (1) in the statistical analysis presented below.

We assumed that the cooperative rearrangement occurs over the entire DCN. Once this rearrangement was completed, the network cluster disappears (dissolved), and the lifetime of the DCN was directly related to the relaxation time (the timescale of the rearrangement). These assumptions may be consistent with the features of the regions responsible for dynamical heterogeneity in supercooled liquids [17]. In addition, the length scale of the heterogeneity is determined by the size of the network, i.e., the number of segments *N* in the DCN. The average size of the DCN was evaluated as the weighted average *N_w_* defined by

(3) $$N_{w}=\frac{\sum_{i}N_{i}^{2}}{\sum_{i}N_{i}}$$

where *N_i_* is the number of segments included in the *i*th DCN. The radius of gyration *R*_g_ of each DCN was evaluated from the simulation as follows:

(4) $$R_{g}=\frac{\sum_{j=1}^{N}{\left[{\left(x_{j}-x_{0}\right)}^{2}+{\left(y_{j}-y_{0}\right)}^{2}+{\left(z_{j}-z_{0}\right)}^{2}\right]}^{1/2}}{N}$$

where (*x_j_*, *y_j_*, *z_j_*) denotes the position of the *j*th correlated segment, and (*x*_0_, *y*_0_, *z*_0_) denotes the position of the center of gravity of the network. *R*_g_ corresponds to the dynamic length scale of the network *ξ*. The fractal dimension *d* was also evaluated based on the scaling relation *N*~*R*_g_*^d^*.

## 3. Results and Discussion

### 3.1. Percolation Transition

**Figure 2:**
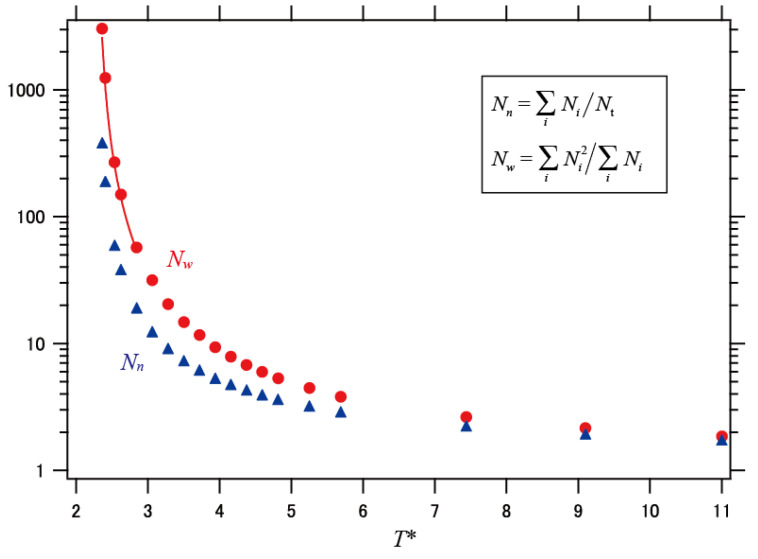
Temperature dependence of the average of the number of segments per DCN. *N_w_* is the weighted average, *N_n_* is the number average, and T* is the reduced temperature defined as T* = *kT/ε*. The solid curve indicates the fitting result based on Equation (5).

Figure 2 shows the *N_w_* plotted against the reduced temperature T*. *N_w_* increased significantly with decreasing temperature, particularly below T* = 3. This behavior suggests a certain critical temperature that corresponds to the percolation. For an ideal Bethe lattice without any constraints, percolation occurs with a coupling probability of 1/(*z* − 1) [36,37]. In the case of *z* = 6, the percolation temperature *T*_p_ is calculated to be 4.481, which is much higher than that apparently seen in Figure 2. The lower *T*_p_ for the present result is due to the condition that loop formation is allowed in the simple cubic lattice, which collapses the network architecture. To evaluate the percolation temperature, we assume a typical scaling law for the critical phenomenon:

(5) $$N_{w}∼\frac{1}{{\left(T*-T_{p}\right)}^{\gamma }}$$

where *γ* is the critical exponent for the percolation transition. We performed a fitting analysis for the profile of *N_w_* in Figure 2 by using Equation (5) near *T*_p_ and determined the best-fit parameters as *γ* = 1.90 and *T*_p_ = 2.29. *T*_p_ may correspond to the critical temperature for an ideal glass transition, where any configurational rearrangement is inhibited. The value of *γ* for an ideal glass transition is not known, but the RFOT predicts that *γ* = 2/*d*, where *d* is the fractal dimension of the cooperative cluster [10]. However, this prediction does not agree well with the present result considering the evaluated d values as shown later (1.55–2.13). The present result indicates a more prominent increase in the cooperativity size on cooling than the prediction from *γ* = 2/*d*.

### 3.2. Geometry of DCN

**Figure 3:**
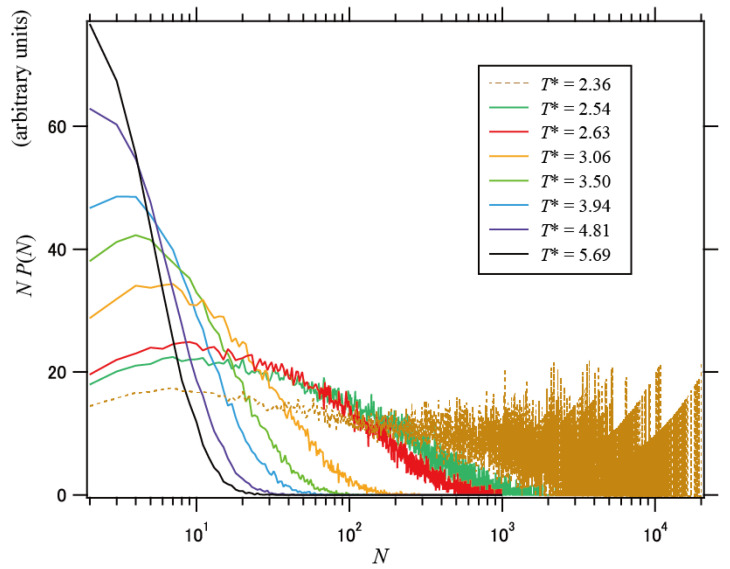
Size distribution profiles of DCNs at various temperatures. The ordinate indicates *N P*(*N*), where *P*(*N*) is the population of the DCN of size *N*.

**Figure 4:**
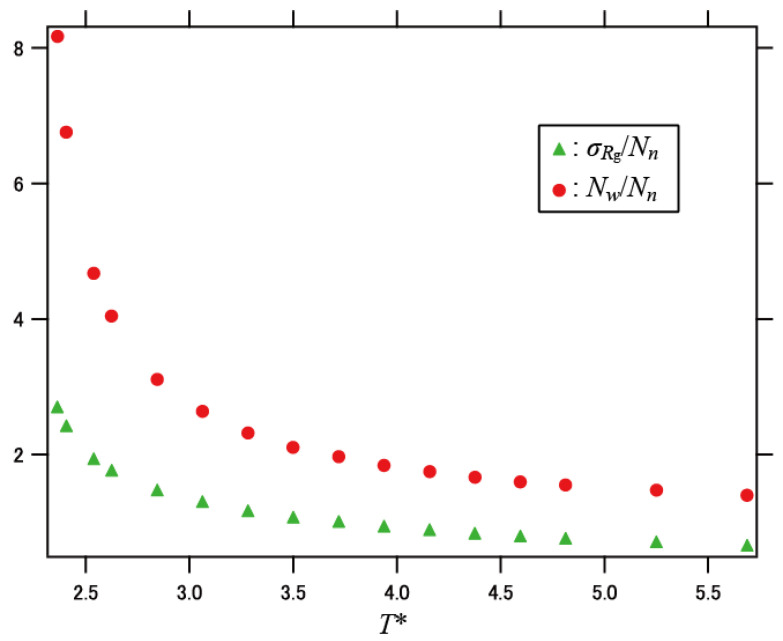
Relative standard deviation *σ*_*R*g_*/N_n_* for the radius of gyration and the dispersion index *N_w_/N_n_* plotted against temperature.

Figure 3 shows the size distribution of the DCNs at different temperatures. *P*(*N*) in Figure 3 is the population of the DCN of size *N*; thus, the profiles of *N* *P*(*N*) denote weighted distributions with respect to *N*. The distribution became broadened with decreasing temperature. This indicates that the distribution of the relaxation times is broadened upon cooling, and that the dynamic heterogeneity becomes significant at lower temperatures. In addition, the relative standard deviation *σ*_*R*g_/*N_n_* for the radius of gyration (*N_n_* is the simple number average of the network size *N*) and the dispersion index *N_w_*/*N_n_* exhibited consistent behaviors, as shown in Figure 4.

As a measure of the geometric feature of the DCN, the fractal dimension *d* of the DCN was evaluated based on *N*~*R_g_^d^*. Typical plots of log *N* vs. log *R*_g_ are presented in Figure S2 in Supplementary Materials. The fractal dimension *d* varies from 1.55 to 2.13 in the temperature range from T* = 5.69 to 2.36. Note that the random walk process leads to *d* = 2. The increase in *d* on cooling indicates that the DCN becomes compact, which is qualitatively consistent with the findings based on the RFOT [38]. As the temperature decreases, the DCN networks become larger and the frequency of loop formation increases. As a result, the network becomes densified at low temperatures, which increases the fractal dimension *d*.

**Figure 5:**
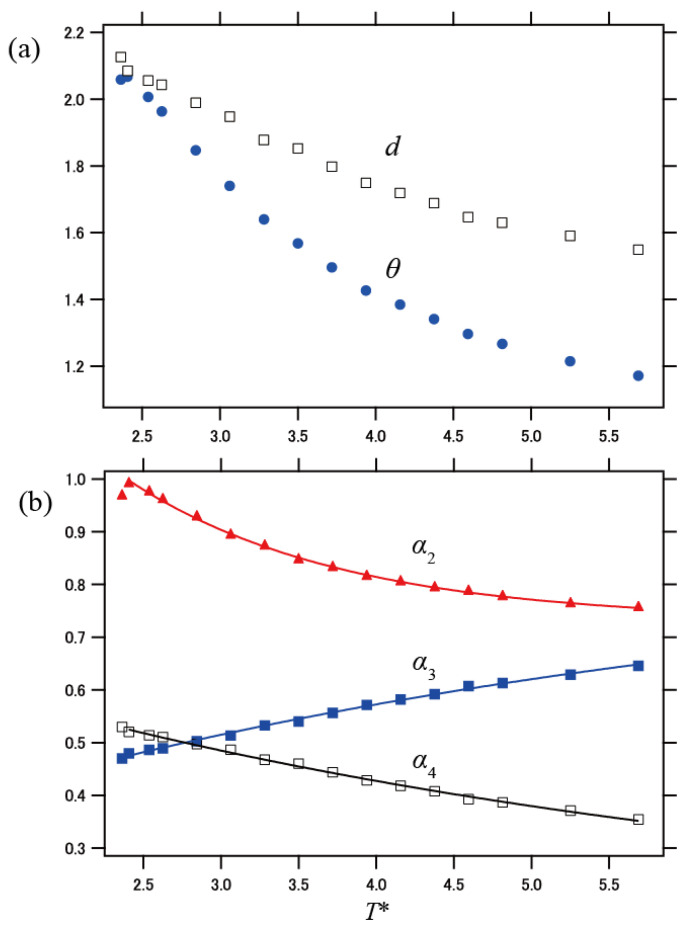
Temperature dependence of exponents; (**a**) *d* and *θ*, and (**b**) *α* for different cases, i.e., *α*_2_ = *θ*/*d*, *α*_3_ = 1/*d*, and *α*_4_ = (*d −* 1)/*d*. The solid curves in (**b**) represent the fitting results based on an empirical expression as described in the Supplementary Materials.

We further evaluated the interfacial (surface) area of the generated DCNs from the number of uncorrelated segments *N*_nc_ that enclose each DCN. *N*_nc_ is the total number of uncorrelated segments adjacent to the outermost segments belonging to the DCN, and we assumed that *N*_nc_ is roughly proportional to the interfacial area of the DCN. Based on a power law for the interfacial area *S~ξ^θ^* and the relation *N*_nc_*~ξ^θ^~R*_g_*^θ^*, the interfacial energy exponent *θ* was estimated. Examples of the plot of log *N*_nc_ vs. log *R*_g_ are shown in Figure S3 in Supplementary Materials. Similar to *d*, *θ* increased with decreasing temperature, as shown in Figure 5a.

At T* = 2.36, *θ* became almost identical to *d*. At low temperatures, densified networks are formed, but they are largely composed of separated strands whose surface area is roughly proportional to the contour length, which is proportional to the number of segments. In this situation, *d* = *θ* is anticipated. On the other hand, at higher temperatures, the majority of DCNs are small clusters, which are not regarded as long strands, resulting in *d* > *θ*. It is expected that at the percolation temperature *T*_p_ (= 2.29), where infinite networks can occur, *d* = *θ* holds.

### 3.3. Segmental Relaxation Time

According to the description of the cooperatively rearranging regions in the Adam–Gibbs and RFOT theories, the relationship between the relaxation time *τ* of the configurational rearrangement and the size of the cooperative region *ξ* is generally expressed as [10]

(6) $$\tau ∼\exp(\Delta \mu \;\xi^{\psi }/kT)$$

where Δ*μ* is the potential energy hindering the segmental rearrangement per segment. Assuming that *ξ~R*_g_~*N_w_*^1/*d*^, we obtain

(7) $$\log\tau =\frac{1}{\ln10}\frac{\Delta \mu }{kT}N_{w}^{\alpha }+\log\tau_{0}$$

where *τ*_0_ is the limiting relaxation time for the high-temperature limit, and *α = ψ*/*d*. The entropy-based theories state that *ψ = d* (Adam–Gibbs), or *ψ = θ* (RFOT). Studies were conducted to explore the values of *ψ* and *θ* so far, although no definite conclusion were reached [24]. For example, a simulation study predicted that *ψ* = 1 and *θ* = 2 [39]. Here we investigate four assumptions for the exponent *α*: (1) *α*_1_ = 1 based on the Adam–Gibbs theory, (2) *α*_2_ = *θ*/*d* based on the RFOT theory, (3) *α*_3_ = 1/*d* which is based on the above simulation result that *ψ* = 1, and (4) *α*_4_ = (*d* − 1)/*d*, which is derived from the maximum value of *ψ* as *ψ* = *d* − 1 [24,40].

We now compare the temperature-dependent relaxation time calculated from Equation (7) with the experimental data reproduced from the literature for the following glass formers: toluene [41], ethylbenzene [42], salol [43], *o*-terphenyl [44], atactic polystyrene (PS) [45,46], polydimethylsiloxane (PDMS) [47], 1,2-polybutadiene (PBD) [46,48], poly(vinyl acetate) (PVAc) [49], and low-molecular-weight poly(methyl methacrylate) (PMMA) [50]. First, we obtained an empirical function for log *N_w_* with respect to T*, and we substituted it into Equation (7). The details are described in the Supplementary Materials. Then, we executed non-linear least squares fitting analysis for the experimental data of segmental relaxation time, where log *τ*_0_, Δ*μ*, and *ε* were treated as the fitting parameters. Note that the dynamical coupling energy *ε* behaves as a temperature-scaling parameter, i.e., *T* = εT*/*k*. The analysis was executed for the four cases of the exponent *α* as mentioned above: *α*_1_ = 1, *α*_2_ = *θ*/*d*, *α*_3_ = 1/*d*, and *α*_4_ = (*d* − 1)/*d*. Based on the results of *d* and *θ* in Figure 5a, temperature-dependent exponent values of *α*_2_, *α*_3_, and *α*_4_ were obtained as shown in Figure 5b. These exponents were introduced in the fitting function of Equation (7) by using empirical functions as described in the Supplementary Materials, and the fitting results are shown by the solid curves in Figure 5b.

**Figure 6:**
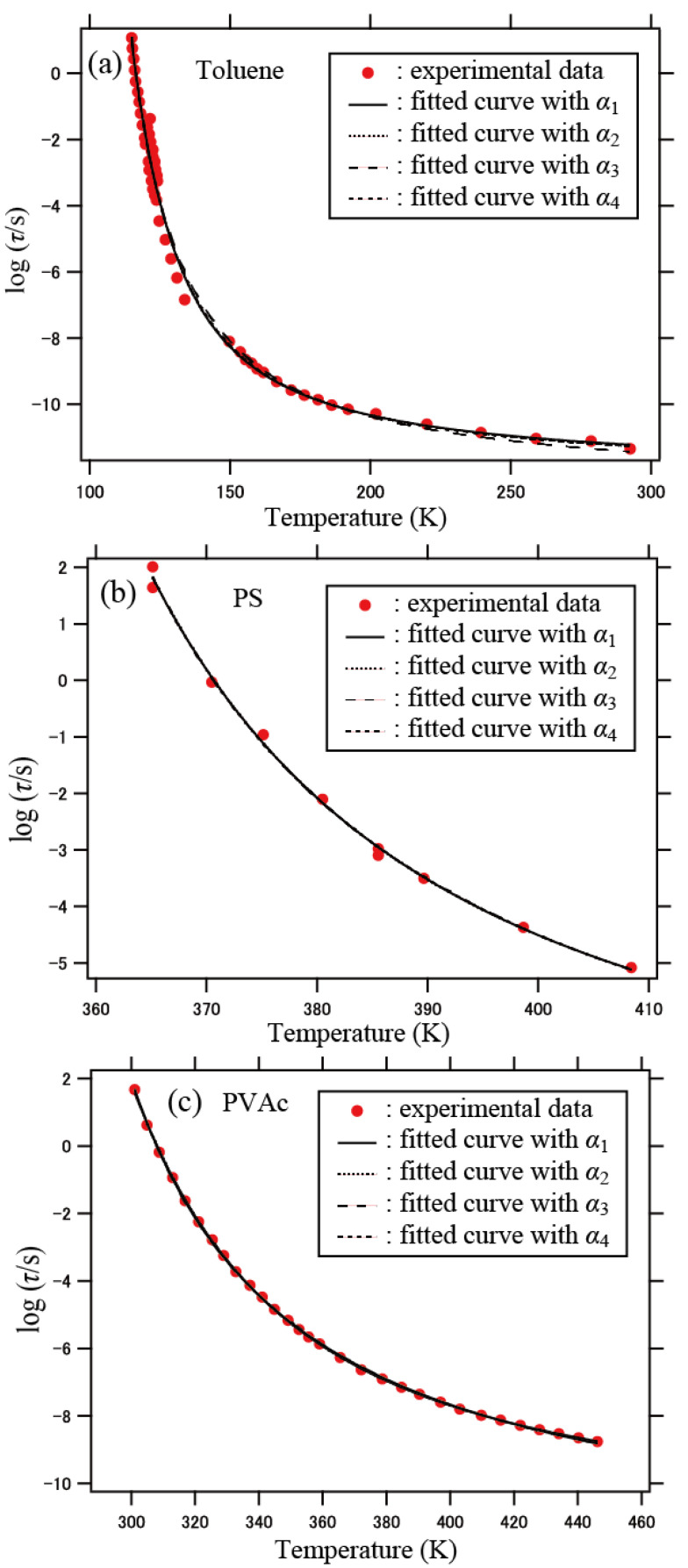
Relaxation time with respect to temperature for (**a**) toluene, (**b**) PS, and (**c**) PVAc. The solid curves are the results of least squares fitting analyses with Equation (7) for *α*_1_, *α*_2_, *α*_3_, and *α*_4_. The experimental data were reproduced from the literature as shown in the text.

The results of the fitting analysis for the relaxation time of toluene, PS, and PVAc are shown in Figure 6. The fitted profiles of the other materials are shown in Figure S4 in Supplementary Materials. The simulation results agreed well with the experimental data for the materials investigated. The parameters obtained for the four cases of *α* are listed in Table 1. Here, the values of the critical temperature *T*_c_ were evaluated from the percolation temperature *T*_p_ as *T*_c_ = (*ε*/*k*)*T*_p_, where *T*_p_ = 2.29, as mentioned previously.

It should be noted that the present DCNs were generated through sequential growth from an initial segment at which the simulation started, and the resulting architecture of the network developed by occasionally forming loops. Thus, in our model, dynamically correlated regions were formed sequentially (not instantaneously). In the DCNs generated via such a sequential process, the actual population of segments *P*(*n*) that have *n* correlated neighbors differs from the equilibrium population that obeys Equation (1), as mentioned previously. The size and geometry of the generated DCNs (and, therefore, their dynamics) reflect the local structural constraint that can inhibit the achievement of the equilibrium (ideal) population of the segments. It may be reasonably understood that the correlated regions develop in a finite time duration, which may be related to the relaxation time for the configurational rearrangements. The agreement with the experimental results shown in Figure 6 suggests that the sequential formation of the cooperatively rearranging regions in supercooled liquids is realistic.

The results also show that the difference in the exponent *α* causes no significant change in the agreement with the experimental data: the differences among the four curves are not apparent in Figure 6. The values of the critical temperature *T*_c_ listed in Table 1 tend to be closer to the values of *T*_0_ (Vogel temperature) for *α*_3_ and *α*_4_ than for *α*_1_ and *α*_2_. Here, *T*_0_ was evaluated from the fitting analysis of the experimental relaxation time *τ* with the Vogel–Fulcher–Tammann function *τ* = *τ*_0_ exp[*B*/(*T* − *T*_0_)], where *B* is a temperature-independent constant [51,52,53]. The above result for *T*_c_ suggests that *α*_3_ and *α*_4_ are more reasonable than *α*_1_ and *α*_2_ for all the materials investigated.

Notably, polymeric materials other than PDMS tend to exhibit high *ε* values (approximately 1 kJ mol^−1^) compared with molecular liquids. This trend may be explained by the interactions between the polymeric segments, including strong covalent bonds, which raise the apparent values of the dynamical coupling energy *ε*. Another characteristic trend of polymeric materials is that the energy barrier for the rearrangement Δ*μ* is relatively low compared with *ε* for *α*_1_ and *α*_2_. In particular, PS and PBD exhibit Δ*μ* < *ε*, or Δ*μ* is approximately equal to *ε* for *α*_1_ and *α*_2_. In contrast, the molecular liquids exhibit Δ*μ* > *ε*.

The fragility parameter *m* defined by [d log *τ*/d(*T*_g_/*T*)]_*T=T*g_ is a measure of the deviation from the Arrhenius law for the relaxation time *τ*. *m* is largely determined by *T*_g_* = (*k*/*ε*) *T*_g_ (*T*_g_ is the laboratory glass transition temperature): the temperature dependence of *N_w_* becomes strong with decreasing T* (Figure 2), and *m* is related to the steepness of *N_w_*. Therefore, *m* is anticipated to have a positive correlation with *ε* and a negative correlation with *T*_g_. This correlation can be verified analytically. From Equation (7), *m* is expressed as

(8) $$m=\frac{\Delta \mu {\left[N_{w}(T_{g}*)\right]}^{\;\alpha }}{k\;T\ln10}\left\{1-\frac{k\;T_{g}}{\varepsilon }\left[\ln N_{w}{\frac{d\alpha }{dT*}|}_{T=T_{g}}+\alpha {\frac{d\ln N_{w}}{dT*}|}_{T=T_{g}}\right]\right\}$$

**Figure 7:**
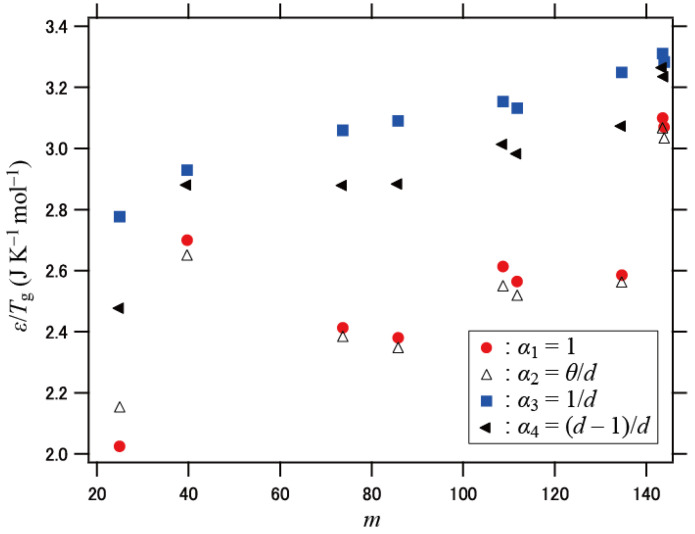
Plots of *ε*/*T*_g_ vs. *m* for the four different assumptions for the exponent *α*.

Note that [*N_w_*(*T*_g_*)]^*α*^ increases with increasing *ε*/*T*_g_, and that the two derivatives in parentheses have a weaker dependence on *ε*/*T*_g_ than *N_w_*(*T*_g_*) in the range of glass transition temperatures. Figure 7 shows *ε*/*T*_g_ for the four cases of the exponent *α* plotted against *m*. Here, the values of *m* were evaluated by fitting the experimental data with the Vogel–Fulcher–Tammann function *τ* = *τ*_0_ exp[*B*/(*T* − *T*_0_)] as *m* = *BT*_g_/[ln 10 (*T* − *T*_0_)^2^] [54]. *T*_g_ was evaluated as the temperature at which *τ* = 10^2^ s. A positive correlation can be observed in Figure 7 for *α*_3_ and *α*_4_. The dispersion of the data is noticeable for *α*_1_ and *α*_2_ compared with *α*_3_ and *α*_4_, which suggests that the assumptions of *α*_3_ and *α*_4_ are more appropriate than those of *α*_1_ and *α*_2_. The above behavior of *m* may be related to the relationship between the fragility and the dynamic length scale at *T*_g_ [29,55,56,57]. The coupling energy *ε*, which denotes the ability of dynamical coupling between segments, can be regarded as the cohesive energy [58]. Thus, the cohesive energy raises the dynamic length scale, which results in a positive correlation between the fragility and the dynamic length scale.

In the present model, we do not explicitly consider any fluctuations in the static structure of the material, i.e., a simple cubic lattice (homogeneous structure) is employed. However, in actual systems, local structural fluctuations may play an important role in determining the dynamics of supercooled liquids. The resulting distributions in the size of the DCN and its geometrical structure originate simply from a stochastic process based on Equation (1), i.e., the fluctuations in energy. Nevertheless, our results are consistent with the experimental data, which indicates that even the fluctuations from the simple stochastic assumption for the energy of segments can mimic the effect of local structural fluctuations on dynamics.

## 4. Conclusions

We proposed a new model that assumes dynamically correlated regions having network shapes. The present model can successfully explain the experimental data for the segmental relaxation time of various glass formers despite the simplicity of the model. The concept of the model is consistent with the notion of cooperative regions with non-compact shapes, such as strings, proposed by simulation studies.

In the simulation process, we employed a simple cubic lattice with *z* = 6, but it is uncertain whether this particular lattice can be applied to a wide variety of glass-forming materials. The applicability may depend on the materials, although at present, there is no reasonable perspective that can predict an appropriate lattice structure specific to individual materials. Nevertheless, the good agreement with the experimental data for various materials appears to be remarkable, which suggests some universality in the features of the dynamics for a wide variety of glass-forming liquids. This universality likely originates from the network-like geometry of the rearranging regions, which penetrate each other in the system. Such a penetrated structure may be inconsistent with the mosaic structure, but it can reasonably explain the mismatch in the length scale between the dynamic heterogeneity and the static fluctuations.

Furthermore, the present model can be extended to materials with nano-confined systems such as ultrathin films, nanofibers, and nanospheres of polymers. The present model has the advantage that the finite-size effects can be easily investigated by changing the size of the lattice in arbitrary directions. Such an attempt is expected to provide valuable insights into the dynamics of nano-confined polymeric systems.

## Figures and Tables

**Table 1:** Summary of the obtained parameters.

Material	Exponent	Δ*μ*/kJ mol^−1^	*ε*/kJ mol^−1^	log (*τ*_0_/s)	*T*_c_/K	*T*_0_/K
Toluene	*α*_1_	1.60	0.28	−11.9	77.8	97
	*α*_2_	2.62	0.28	−12.1	76.7	
	*α*_3_	2.88	0.36	−12.6	98.7	
	*α*_4_	3.89	0.34	−12.2	92.8	
Ethylbenzene	*α*_1_	1.30	0.30	−11.7	82.0	102
	*α*_2_	2.08	0.29	−11.8	81.1	
	*α*_3_	2.36	0.37	−12.0	103.0	
	*α*_4_	3.19	0.35	−11.9	97.4	
Salol	*α*_1_	2.31	0.58	−12.1	159.2	189
	*α*_2_	4.09	0.56	−12.5	155.1	
	*α*_3_	5.79	0.70	−13.7	192.1	
	*α*_4_	7.00	0.67	−12.9	183.6	
*o*-Terphenyl	*α*_1_	3.08	0.63	−12.5	172.5	209
	*α*_2_	5.20	0.61	−12.7	169.1	
	*α*_3_	6.88	0.76	−13.6	210.7	
	*α*_4_	8.70	0.73	−13.1	200.6	
PS	*α*_1_	0.65	1.13	−8.1	312.1	335
	*α*_2_	0.97	1.12	−8.1	308.2	
	*α*_3_	5.11	1.21	−10.5	333.3	
	*α*_4_	4.59	1.19	−9.4	328.6	
PDMS	*α*_1_	2.86	0.30	−2.9	81.5	101
	*α*_2_	3.92	0.31	−3.1	86.5	
	*α*_3_	3.85	0.41	−3.3	111.8	
	*α*_4_	6.44	0.36	−3.3	99.7	
PBD	*α*_1_	0.57	0.83	−8.5	229.9	248
	*α*_2_	0.85	0.82	−8.3	226.9	
	*α*_3_	4.38	0.89	−11.1	245.9	
	*α*_4_	3.94	0.88	−9.9	242.3	
PVAc	*α*_1_	5.05	0.71	−11.5	196.8	249
	*α*_2_	8.38	0.70	−11.8	193.8	
	*α*_3_	8.19	0.93	−12.2	255.6	
	*α*_4_	11.4	0.87	−11.7	238.5	
PMMA (low M_w_)	*α*_1_	1.14	0.99	−8.1	81.5	298
	*α*_2_	1.88	0.97	−8.0	86.5	
	*α*_3_	7.43	1.08	−11.3	111.8	
	*α*_4_	6.92	1.06	−9.8	99.7	

References of the experimental data used in the analysis are presented in the text.

## Supplementary Materials

The following are available online at https://www.mdpi.com/2073-4360/13/19/3424/s1, Figure S1: The probability of dynamical coupling with neighboring segments, Figure S2: Plot of log *N* vs. log *R*_g_ at *T** = 3.063 and 4.376, Figure S3: Plot of log *N*_nc_ vs. log *R*_g_ at *T** = 3.063 and 4.376, Figure S4: Relaxation time with respect to temperature for ethylbenzene, salol, *o*-terphenyl, PDMS, PBD, and low M_w_ PMMA, Table S1: Obtained fitting parameters in Equation (S2).
