# Supplementary material for: A Dynamically Correlated Network Model for the Collective Dynamics in Glass-Forming Molecular Liquids and Polymers
Source: Polymers (Basel). 2021 Oct 6;13(19):3424. doi: 10.3390/polym13193424 (PMC8512962; doi:10.3390/polym13193424)
Supplement: Supplementary file 1 [file polymers-13-03424-s001.zip › polymers-1406435-SI.pdf]

Supplementary Materials

Dynamically correlated network model for the collective dynamics in glass-forming molecular liquids and polymers

Takashi Sasaki*, Yuya Tsuzuki, Tatsuki Nakane

Department of Materials Science and Engineering, University of Fukui, Fukui 9108507 Japan
*sasakita@u-fukui.ac.jp

1. The probability of dynamical coupling

The probabilities of the dynamical coupling with neighboring segments, which were calculated according to Equation (1) in the main text, are plotted against reduced temperature in Figure S1. Here, the coordination number z is 6, which is the case for a simple cubic lattice.

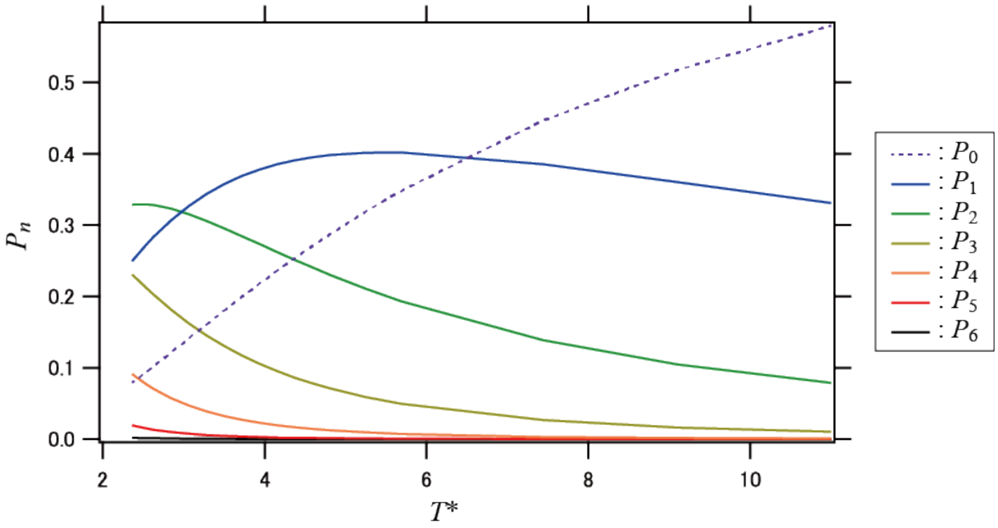

Figure S1. Probabilities of dynamical coupling with neighboring segments.

2. Evaluation of fractal dimension

The fractal dimension d of the generated DCNs were estimated according to the power law $N \sim R_g^{\,d}$, where N is the number of segments in the DCN, and R_g is the radius of gyration of the DCN. Typical plots of log N vs. log R_g are shown in Figure S2. As for the surface area of the DCN, we also evaluated the exponent θ according to the power law

$N_{nc} \sim \xi^{\theta} \sim R_g^{\theta}$. Here, N_{nc} is the number of uncorrelated segments that enclose the DCN. Typical plots of log N_{nc} vs. log R_g are shown in Figure S3.

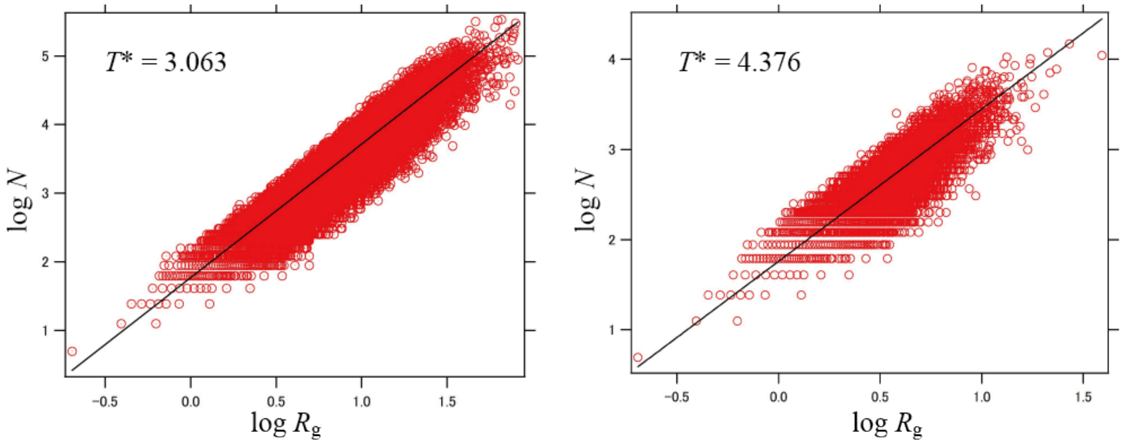

Figure S2. Plots of log N vs. log R_g at T^* = 3.063 and 4.376. Each plot includes data points of 10^5 DCNs. The solid lines indicate the results of linear regression analysis.

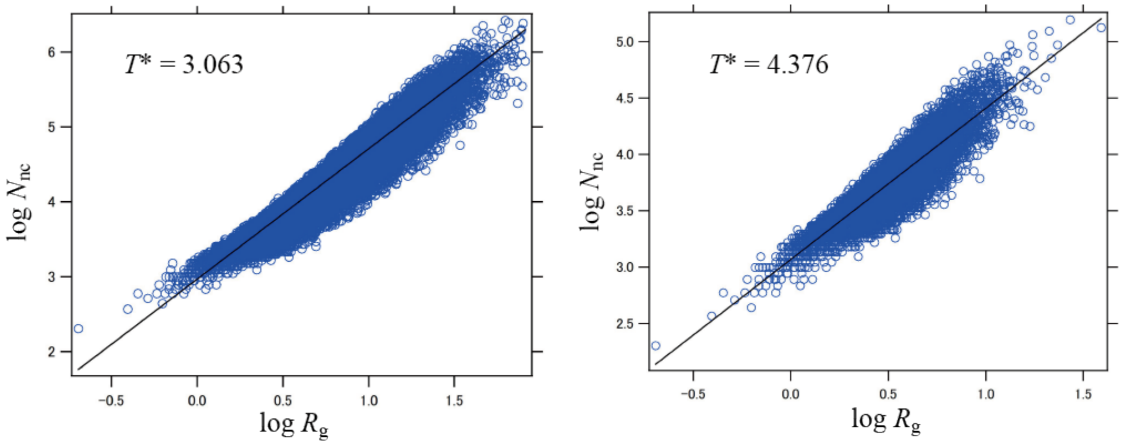

Figure S3. Plots of log N_{nc} vs. log R_g at T^* = 3.063 and 4.376. Each plot includes data points of 10^5 DCNs. The solid lines indicate the results of linear regression analysis.

3. Fitting analysis of the relaxation time

To compare the simulation results with the experimental data, we first obtained an empirical function for log N_w (N_w is the weight average of the number of segments N in the DCN) as

$$\log N_w = c\{b_1 \exp[-(T^* - T_0)/T_1] + b_2 \exp[-(T^* - T_0)/T_2] + (1 - b_1 - b_2)\exp[-(T^* - T_0)/T_3]\} \qquad \text{(S1)}$$

where b_1, b_2, T_0, T_1, T_2, T_3, and c are fitting parameters to emulate the master curve of log N_w vs. T^* profile. Note that equation (S1) shows $\lim_{T^*\to\infty} N_w = 1$, that is, no collective motions occur at the high temperature limit. Non-linear least squares fitting analysis yielded the best-fit parameters as b_1 = 0.14292, b_2 = 0.28894, T_0 = 2.1828, T_1 = 6.7363, T_2 = 0.82917, T_3 = 0.13495, and c = 6.6612. We also evaluated empirical functions for the temperature-dependent exponent α for α_2, α_3, and α_4 as

$$\alpha(T^*) = b_0 + b_1 \exp[-(T^* - T_0)/T_1] + b_2 \exp[-(T^* - T_0)/T_2] \qquad \text{(S2)}$$

where b_0, b_1, b_2, T_1, and T_2 are fitting parameters. The best-fit parameters obtained via non-linear least squares fitting analysis are listed in Table S1. T_0 was fixed to 2.407. Note that α_3 exhibits a symmetrical shape with that of α_4, as can be derived from the definition.

Table S1. Obtained fitting parameters in Equation (S2).

α	b_0	b_1	b_2	T_1	T_2
α_2	0.73138	0.13721	0.12867	1.328	1.407
α_3	0.85333	–0.19002	–0.18787	6.1402	4.4413
α_4	0.14667	0.19002	0.18787	6.1402	4.4413

The results of the fitting analysis for the experimental data of relaxation time for ethylbenzene, salol, *o*-terphenyl, PDMS, PBD, and low M_w PMMA are shown in Figure S4. In general, fairly good agreements were obtained for these materials, although *o*-terphenyl exhibited small deviations at higher temperatures. Salol also showed small deviations in the entire range of temperature.

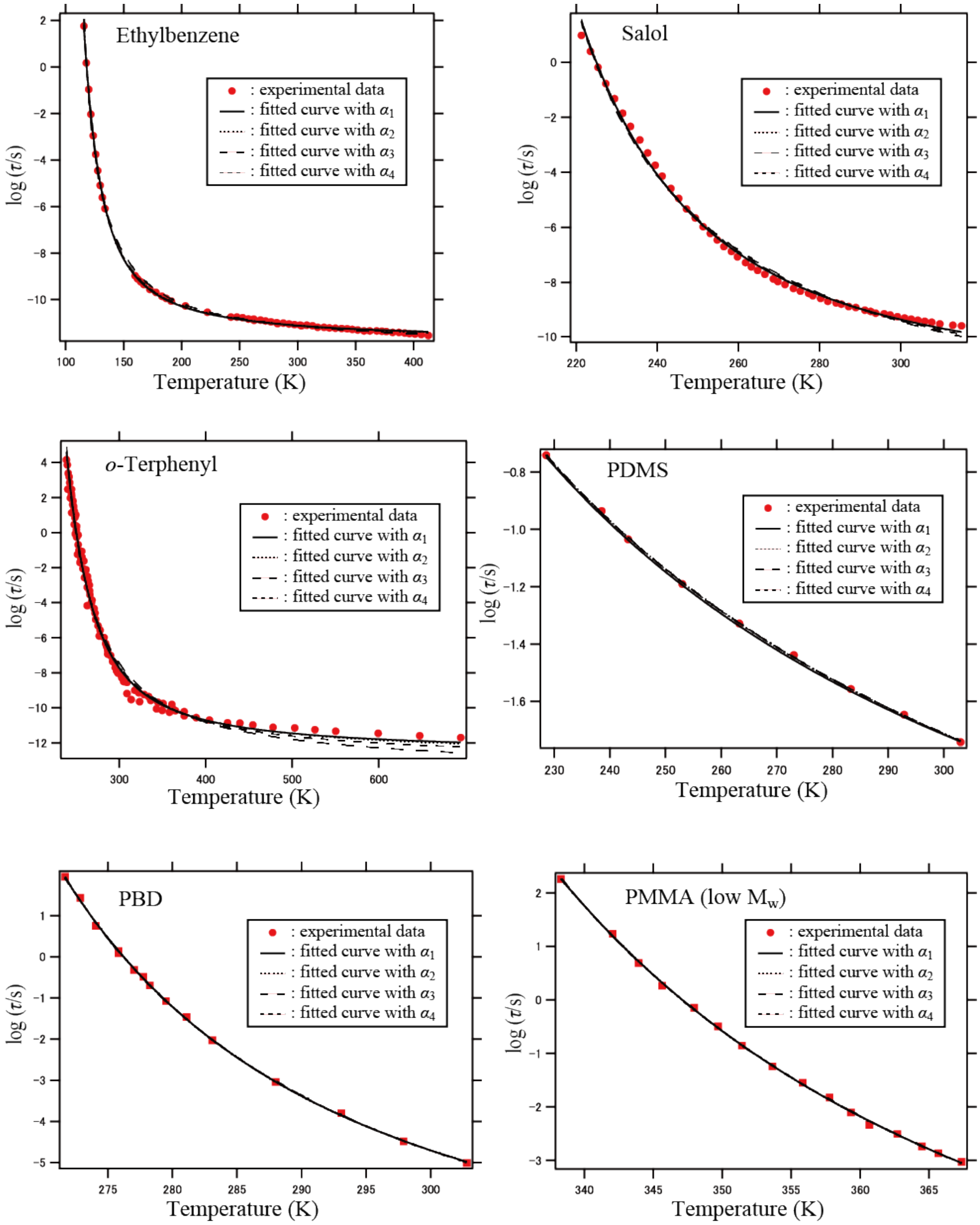

Figure S4. Relaxation time with respect to temperature for ethylbenzene, salol, *o*-terphenyl, PDMS, PBD, and low M_w PMMA. The solid curves are the results of least squares fitting analyses with Equation (7) in the main text for α_1, α_2, α_3, and α_4.
